# Adherence to β-hydroxy-β-methylbutyrate-Enriched Oral Nutritional Supplements Enhances Survival and Nutritional Recovery in Malnourished Outpatients: Prognostic Insights

**DOI:** 10.3390/nu17091601

**Published:** 2025-05-07

**Authors:** Isabel Vegas-Aguilar, Rocío Fernández-Jiménez, Isabel Cornejo-Pareja, María Del Mar Amaya-Campos, Patricia Guirado-Peláez, Natalia Montero-Madrid, Álvaro Vidal-Suarez, Maria Angeles Martín-Fontalba, Victor Simon-Frapolli, Francisco J. Tinahones, José Manuel García-Almeida

**Affiliations:** 1 Department of Endocrinology and Nutrition, Virgen de la Victoria University Hospital, 29010 Málaga, Spain; isabel.mva13@gmail.com (I.V.-A.); isabelmaria_cornejo@hotmail.com (I.C.-P.); mariadelmarac2@gmail.com (M.D.M.A.-C.); pguirado1991@gmail.com (P.G.-P.); nataly_montero@hotmail.com (N.M.-M.); alvarovidal1992@gmail.com (Á.V.-S.); mamfontalba28@gmail.com (M.A.M.-F.); victorsimonfrapolli.med@gmail.com (V.S.-F.); fjtinahones@hotmail.com (F.J.T.); jgarciaalmeida@gmail.com (J.M.G.-A.); 2 Instituto de Investigación Biomédica de Málaga y Plataforma en Nanomedicina—IBIMA Plataforma BIONAND, 29010 Málaga, Spain; 3 Department of Medicine and Dermatology, Málaga University, 29016 Malaga, Spain; 4 Department of Endocrinology and Nutrition, Quironsalud Málaga Hospital, Av. Imperio Argentina, 29004 Malaga, Spain; 5 Centro de Investigación Biomédica en Red de Fisiopatología de la Obesidad y Nutrición (CIBEROBN), Instituto de Salud Carlos III, 28029 Madrid, Spain

**Keywords:** malnutrition, dietary supplements, muscle strength, nutritional status, prognosis

## Abstract

**Background**: Disease-related malnutrition (DRM) in outpatients is associated with increased mortality and functional decline. Morphofunctional assessments, including phase angle (PA), rectus femoris cross-sectional area (RF-CSA), and handgrip strength (HGS), provide valuable prognostic insights in the ambulatory setting. Nutritional recovery programs enriched with β-hydroxy-β-methylbutyrate (HMB) offer potential benefits in improving nutritional and functional outcomes. **Objective**: To evaluate the effects of a six-month nutritional recovery program combining HMB-enriched oral nutritional supplements (HMB-ONS), dietary recommendations, and exercise on survival, morphofunctional markers, and adherence in malnourished outpatients. **Methods**: This retrospective observational study included 135 malnourished outpatients diagnosed using GLIM criteria. Morphofunctional assessments included PA (bioimpedance analysis), RF-CSA (nutritional ultrasound), HGS (dynamometry), and the Timed Up and Go (TUG) test. Adherence was assessed using pharmacy retrieval records and a validated questionnaire. Changes in morphofunctional markers and their association with mortality were analyzed using multivariate Cox regression models. **Results**: After six months, significant improvements were observed in PA (+0.47°), RF-CSA (+0.90 cm^2^), HGS (+4.1 kg), and TUG (−0.93 s) (all *p* < 0.001). These improvements were more pronounced in the high-adherence group, which also exhibited a reduced mortality risk (HR 0.42, *p* < 0.05). Changes in PA and HGS were strongly associated with survival, with ΔPA showing an HR of 0.27 (95% CI: 0.15–0.50, *p* < 0.001) and ΔHGS showing an HR of 0.82 (95% CI: 0.75–0.89, *p* < 0.001). **Conclusions**: A nutritional recovery program with HMB-ONS significantly improves survival and morphofunctional markers in malnourished patients, with the greatest benefits observed in those with high adherence. These findings underscore the importance of adherence-support strategies in optimizing clinical outcomes and highlight the need for further research to confirm long-term benefits.

## 1. Introduction

Disease-related malnutrition (DRM) is a widespread health issue associated with increased mortality, particularly in vulnerable populations such as the elderly and those with chronic diseases, such as cancer [1]. Poor nutritional status weakens the immune system, impairs wound healing, and exacerbates the risk of infection, all of which contribute to higher mortality rates [2,3]. The most widely accepted diagnostic criteria for DRM include the Subjective Global Assessment (SGA) and the Global Leadership Initiative on Malnutrition (GLIM) criteria [4]. Muscle loss, also related to sarcopenia, is a critical component in the nutritional assessment of patients with chronic conditions [5]. In this context, morphofunctional assessments have emerged as essential tools for a comprehensive evaluation of body composition and function in malnourished patients [6]. These techniques include vectorial bioelectrical impedance analysis (BIVA) to measure phase angle (PA), nutritional ultrasound to assess muscle quantity and quality, functional tests, and dynamometry to evaluate muscle strength [7]. These methods provide a more accurate picture of the nutritional status of the malnourished patients, beyond traditional indicators [8]. Therefore, addressing malnutrition is critical for improving patient outcomes. Once malnutrition is detected, it becomes crucial to determine the specific nutritional intervention needed [9]. One potential intervention, which also scores well for adherence, is the use of nutritional supplements such as high-calorie protein oral nutrition supplement (ONS) [10], as well as other supplements, such as β-Hydroxy-β-methyl-butyrate (HMB). HMB is an active metabolite of leucine, which is one of the three essential branched-chain amino acids [11]. HMB-enriched supplements have been shown to improve prognosis in malnourished patients by promoting muscle mass recovery and reducing the risk of complications related to malnutrition, as evidenced by findings from the NURISH study [12]. 

Recent evidence indicates that while both standard and HMB-enriched oral nutritional supplements (ONS) can improve nutritional and liver function parameters, HMB provides additional benefits, such as enhanced muscle strength and reduced minimal hepatic encephalopathy in cirrhotic patients [13]. Moreover, HMB-enriched high-protein ONS have demonstrated promising effects in mitigating muscle loss during hospitalization and rehabilitation [14]. Long-term use of specialized ONS containing HMB has also been associated with improvements in biochemical and hematological markers in community-dwelling older adults at risk of malnutrition [15], supporting the use of HMB as part of nutritional strategies for disease-related malnutrition (DRM). In cancer patients with cachexia, therapeutic doses of HMB have been shown to improve muscle strength and physical performance [16]. Notably, a double-blind, randomized, placebo-controlled clinical trial found that a high-protein, HMB-enriched supplement significantly reduced post-discharge mortality in older patients hospitalized for chronic conditions, highlighting the prognostic value of nutritional interventions that extend beyond traditional clinical outcomes [12].

However, while nutritional supplements are widely recommended, their long-term effects on recovery, muscle quantity, and mortality are not well understood. The present study aims to evaluate the effect of a six-month nutritional recovery program with dietary recommendations, exercise, and HMB-enriched specific oral supplements (HMB-ONS) in patients with malnutrition after six-months of follow-up. We also aim to investigate the effect of ONS in BIVA, muscle quantity and quality, and their relationship with mortality. Finally, we assess whether HMB-ONS are able to recover patients with higher GLIM value and with sarcopenia, after 6 months of nutritional supplement program.

## 2. Materials and Methods

### 2.1. Study Design and Patients Included in the Study

This was a retrospective observational study conducted at the Clinical Nutrition Unit of Virgen de la Victoria University Hospital (Málaga, Spain), aimed at evaluating the impact of a six-month HMB-enriched nutritional recovery program on morphofunctional parameters and survival in patients with disease-related malnutrition (DRM), under the hypothesis that combined nutritional and functional interventions would lead to improvements in body composition, physical performance, and clinical outcomes. Eligible participants were adults aged 18–85 years with a diagnosis of DRM according to the GLIM criteria, referred from various specialties (e.g., oncology, gastroenterology, neurology, internal medicine) for outpatient nutritional assessment, including those with cancer (under chemotherapy, radiotherapy, or post-surgical recovery), inflammatory bowel disease, neuromuscular or autoimmune conditions, and other chronic diseases associated with malnutrition (Supplementary Table S1). Inclusion criteria included the following, capacity and willingness to follow the six-month nutritional recovery program (including oral nutritional supplementation, dietary counseling, and exercise), and attendance at regular follow-up visits. Exclusion criteria included pregnancy, contraindications to bioelectrical impedance vector analysis (BIVA) or nutritional ultrasound. The patient flowchart is summarized in Supplementary Figure S1.

### 2.2. Nutritional Assessment

All patients met malnutrition criteria based on the GLIM and participated in a six-month nutritional recovery program consisting of HMB-ONS personalized dietary recommendations, an adapted exercise plan, and 12–24 months of follow-up for mortality prognosis. The nutritional plan was completely detailed elsewhere [17]. Briefly, a trained dietitian provided tailored recommendations to enhance diet quality and physical activity aimed at muscle strengthening. These recommendations included caloric and protein enrichment strategies. The recommended daily energy intake was 30–35 kcal per kg of body weight (BW), while protein intake was set at 1.2–1.3 g/kg BW. Patients were instructed to supplement with two servings of an ONS daily. The ONS provided 1.5 kcal/mL, delivering 330 kcal, 20 g protein, 11 g fat, 37 g carbohydrates, 1.7 g fiber, 1.5 g calcium HMB, and 500 IU vitamin D per 220 mL serving (Ensure^®^ Plus Advance, Abbott Nutrition, Spain). Dietary intake and adherence were monitored using the 24-hour recall method over three days, conducted through patient interviews by the dietitian. Adherence and tolerance to the ONS were carefully evaluated throughout the study.

### 2.3. Morphofunctional Assessment 

#### 2.3.1. Bioelectrical Impedance Vectoral Analysis

A body composition study was carried out using bioelectrical impedance analysis (BIVA) and nutritional ultrasound. Muscle strength was evaluated through dynamometry, while functionality was assessed using the Up and Go test. BIVA was performed using a 50 kHz phase-sensitive impedance analyzer (Whole Body Bioimpedance Vector Analyzer, Nutrilab, AKERN, Florence, Italy) with tetrapolar electrodes delivering 800 μA. Electrodes were positioned on the right hand and foot, and measurements were taken after the patient rested in a supine position for 5 min to stabilize fluid shifts. Various parameters were recorded, including phase angle (PA, °), standardized phase angle (SPA), body cell mass (BCM, kg), body cell mass index (BCMI, kg/m^2^), fat mass (FM, kg), fat mass index (FMI, kg/m^2^), fat-free mass index (FFMI, kg/m^2^), appendicular skeletal muscle mass (ASMM, kg), skeletal muscle index (SMI, kg/m^2^), total body water (TBW, kg), extracellular water (ECW, kg), intracellular water (ICW, kg), hydration levels (Hydragram^®^, % (TBW/FFM)), nutrition levels (Nutrigram^®^ [mg/24 h/m) reactance (Xc, Ω/m), and resistance (Rz, Ω/m). Height was measured with a Seca stadiometer (Hamburg, Germany). The BIVA measurements were standardized by sex and age, based on data from healthy Italian adults. The PA was calculated as arctan (Xc/Rz) × (180°/π). The SPA for each participant was determined by subtracting the reference PA from the observed PA and dividing by the reference standard deviation (SD) for age and sex. The accuracy of the BIVA device was checked daily using a precision circuit provided by the manufacturer (AKERN, Florence, Italy), consistently producing values near the reference value of 385 Ohms. In vivo reproducibility of BIVA measurements showed coefficients of variation (CV) of 1–2% for Rz and Xc.

#### 2.3.2. Ultrasound Muscle Measurement

Muscle ultrasound of the rectus femoris in the quadriceps (QRF) was performed on all participants using a 10–12 MHz probe with a multifrequency linear array (Mindray Z60, Madrid, Spain) while they were in a supine position. The measurement was conducted without compression at the lower third between the patella’s superior pole and the anterior superior iliac spine, capturing the muscle thickness, circumference, and cross-sectional area. A trained physician performed the ultrasound, with the probe positioned perpendicular to the QRF’s longitudinal and transverse axes to measure the rectus femoris cross-sectional area (RF-CSA), circumference (RF-CIR), axes (*X*-axis and *Y*-axis), and leg subcutaneous fat (L-SAT). Three measurements were taken for each parameter, with the mean value calculated. For abdominal adipose tissue evaluation, the midpoint between the xiphoid process and the navel was measured to assess total subcutaneous abdominal fat (T-SAT), superficial subcutaneous abdominal fat (S-SAT), and visceral fat (VAT). 

#### 2.3.3. Functional Measurement

Handgrip strength (HGS) was measured using a Jamar hand dynamometer (Asimow Engineering Co., Los Angeles, CA, USA) in a seated position with the elbow flexed at 90° on the dominant hand. Participants performed three maximal isometric contractions, with short pauses in between, and both the maximum and average values were recorded. Functional capacity was evaluated using the Timed Up and Go test, measuring the time in seconds to rise from a chair, walk 3 meters, turn around, walk back, and sit down.

### 2.4. Adherence to ONS

To assess adherence in this study, a combination of direct and indirect methods was used. Adherence was first evaluated directly by calculating the percentage of prescribed HMB-ONS retrieved from the hospital pharmacy, with patients classified as having high adherence if they collected ≥75% of the prescribed supplements, and low adherence if below this threshold. Indirectly, adherence was further measured using the validated adherence questionnaire by Wanden-Berghe et al. (2018) [18], which assesses two dimensions: knowledge and difficulties. The questionnaire includes six dichotomous items (yes/no), scored based on patient familiarity with the purpose, brand, and intake method of their nutrition, as well as reported intake barriers (e.g., skipped doses, schedule changes). Patients were classified into low-, moderate-, or high-adherence categories according to their cumulative score on the questionnaire. This dual-method approach allowed for a robust assessment of adherence, considering both objective data from pharmacy records and patient-reported adherence behavior. Patients are classified as adherent if they show favorable results in both adherence measurement techniques.

### 2.5. Nutritional Diagnosis

To diagnose malnutrition based on GLIM criteria, both a phenotypic and an etiologic criterion were required. The phenotypic criteria for diagnosing moderate malnutrition (criteria 1) included the following: weight loss of 5–10% over the past 6 months, a BMI < 20 kg/m^2^ for individuals under 70 years old or <22 kg/m^2^ for those 70 and older, or a fat-free mass index (FFMI) < 17 kg/m^2^ in men or <15 kg/m^2^ in women. Severe malnutrition (criteria 2) was defined by weight loss exceeding 10%, and a BMI < 18.5 kg/m^2^ for those younger than 70 years or <20 kg/m^2^ for those aged 70 or above. The etiologic criteria included reduced food intake/assimilation and the burden of disease/inflammation. Reduced intake was identified when food consumption was less than 50% of calculated needs, calculated based on quartiles of intake over the last 5 days. Gastrointestinal symptoms such as dysphagia, nausea, vomiting, diarrhea, constipation, and abdominal pain were assessed as indicators of poor assimilation. In patients with active tumors requiring radiotherapy, all were considered to meet the inflammation criterion. The main clinical outcomes included the mortality rate at 24 months. Disease status was determined by the attending physician, and clinical outcomes were retrieved from the hospital’s medical records.

### 2.6. Statistical Analysis

The results are reported as mean ± standard deviation (SD) for continuous variables and as numbers (percentages) for categorical variables. Significant differences between groups are indicated by an asterisk, based on the ANOVA test followed by pairwise comparisons with adjustments for multiple testing using the Benjamini–Hochberg method. The chi-squared test was applied for percentage-based variables. Logistic regression analysis was used to evaluate the association between body composition and functionality parameters with the severity of malnutrition, and the odds ratio (OR) with 95% confidence intervals (CI) was calculated. The results were adjusted for age, sex, and BMI (categorized as BMI < 20 for patients younger than 70, and BMI < 22 for patients 70 years or older). The predictive ability of muscle mass variables for malnutrition was evaluated using the receiver operating characteristic (ROC) curve and the area under the curve (AUC). Multivariable Cox regression was employed to predict mortality risk, utilizing the rms and foreign packages in R software. The follow-up has a median of 730 days (2 years), with an interquartile range of 365–1095 days (1–3 years), designed to assess overall mortality. A decision tree was created using the *rpart* package, and a Random Forest analysis was conducted with the *RandomForest* package, with graphical representations generated using R software v. 3.5.1 (RStudio, PBC, Boston, MA, USA). Statistical significance was determined by a *p*-value of <0.05.

## 3. Results

### 3.1. General Baseline Characteristics of the Patients Included in the Study

At baseline, a total of 135 patients complies eligibility criteria, age mean 61.3 years (SD: 14.7) and females 55.6% (Table 1). The mean BMI was 22.0 kg/m^2^ (SD: 3.83). Body composition according to BIVA showed the following: PA mean of 4.93° (SD: 1.02) and SPA mean of −0.22 (SD: 1.49). BCM mean of 22.3 kg (SD: 6.29) and BCMI of 8.10 (SD: 1.83). FFM was 46.6 kg (SD: 8.92) and FFMI of 17.0 (SD: 2.17). FM was 13.4 kg (SD: 7.02) and FMI of 4.95 (SD: 2.70). Ultrasound muscle mass assessment quantity showed RF-CSA mean of 3.17 cm^2^ (SD: 1.26) and RF-CIRC, mean 8.31 cm (SD: 1.39). In addition, RF-*X*-axis and RF-*Y*-axis reported means of 3.58 cm (SD: 0.61) and 1.02 cm (SD: 0.31), respectively. Functional parameters included handgrip strength, with a mean of 24.3 kg (SD: 9.73), and the Timed Up and Go (TUG) test, with a mean completion time of 7.54 seconds (SD: 1.97). The remaining variables regarding adipose tissue and hydration are summarized in Table 1.

At baseline, men showed significantly higher values than women across several parameters, such as in BIVA analysis (*p* < 0.05), although women showed a higher fat mass index (5.66 vs. 4.07, *p* = 0.002). Nutritional ultrasound parameters also revealed significantly higher values in men (*p* < 0.05). In terms of functional parameters, men displayed higher handgrip strength (30.4 kg vs. 19.4 kg, *p* < 0.001). For water content, extracellular water (20.0 kg vs. 16.0 kg, *p* < 0.001) and total body water (40.0 kg vs. 30.3 kg, *p* < 0.001) were also greater in men (Table 1).

### 3.2. Changes in Morphofunctional Assessment After the Nutritional Intervention

After nutritional intervention, a significant improvement in anthropometric, BIVA, muscle mass functional assessment, and nutritional status variables was observed, all summarized in Table 2. Weight and BMI significantly increased from a baseline (*p* < 0.001, respectively). As for the BIVA, PA and SPA significantly improved from the baseline (*p* = 0.010, and *p* = 0.008, respectively). Furthermore, BCM and BCMI increased from 22.29 (SD: 6.29) to 23.22 (SD: 6.88) (*p* = 0.010) and from 8.10 (SD: 1.83) to 8.42 (SD: 2.03) (*p* = 0.009), respectively. FFM and FFMI as well as FM and FMI significantly increased from the baseline (*p* = 0.009, *p* = 0.013, *p* = 0.004, and *p* = 0.004, respectively). Both total body water and Nutrigram by BIVA showed a significant rise from the baseline (*p* = 0.023 and *p* = 0.010, respectively). Finally, as for ultrasound measurements, RF-CSA, RF-CIRC, RF-*Y*-axis, and L-SAT, as well as HGS, significantly improved from the baseline (all with *p* < 0.001).

### 3.3. Impact of Nutritional Intervention on Malnutrition and Muscle Loss

**Figure 1:**
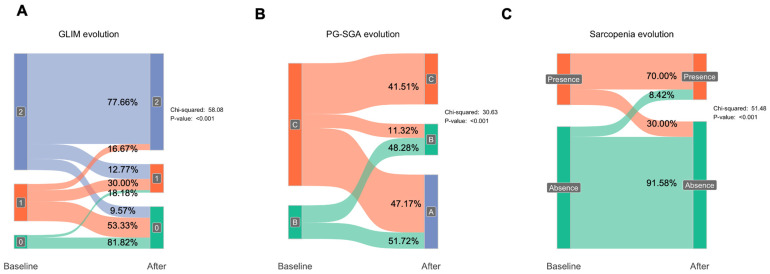
Mosaic plot showing diagnostic improvement in malnourished patientsanel (**A**) shows shifts in Global Leadership Initiative on Malnutrition (GLIM) criteria following the nutritional intervention, with patients moving from severe (criteria 2) to moderate (criteria 1) or no malnutrition (criteria 0). Panel (**B**) illustrates changes in Subjective Global Assessment (SGA) categories, highlighting recovery from more severe states (C to B or A). Panel (**C**) highlights the percentage of sarcopenic patients who recovered after the intervention. Statistical significance is denoted for observed changes where applicable. **Abbreviations: GLIM**: Global Leadership Initiative on Malnutrition. **SGA**: Subjective Global Assessment.

After a six-month follow-up period, nutritional intervention was assessed using GLIM and PS-SGA. Regarding GLIM, 16.67% of those classified under “GLIM criteria 2” (Severe malnutrition) changed to criteria 1 (Moderate malnutrition), and 9.57% improved to criteria 0 (No malnutrition) (Figure 1). Among patients in criteria 1, 53.33% returned to criteria 0, while 30% remained in criteria 1 and 16.67% moved to criteria 2. For those initially classified under criteria 0, 81.82% remained in criteria 0 while 18.18% moved to criteria 1 (*p* < 0.001). Regarding the PG-SGA, 51.72% of patients in criteria B improved to criteria A, and 48.28% of patients remained in the same criteria. For patients in criteria C, 41.51% remained in the same criteria, while 11.32% moved to criteria B and 47.17% moved to criteria A (*p* < 0.001). For sarcopenia, 30% of sarcopenic patients recovered from sarcopenia, and only 8.42% became sarcopenic (*p* < 0.001).

### 3.4. Impact of the Adherence to the Nutritional Supplements in Patients Included in the Study

The following results are presented for all groups, with comparisons between those with low adherence and high adherence, along with the *p*-value for significance. There is no significant difference in educational level, socioeconomic status, or comorbidities between patients with high and low adherence. We observed that the low-adherence group had a slightly higher PA and SPA when compared to the high-adherence group (*p* = 0.045 and *p* = 0.003, respectively) (Supplementary Table S2). Furthermore, the low-adherence group had lower FM and FMI than the high-adherence group (*p* = 0.023 and *p* = 0.028, respectively). Finally, the low- adherence group had a faster Up and Go in comparison with the high-adherence group (*p* = 0.007), showing a significant difference in functionality.

### 3.5. Changes in Morphofunctional Assessment After the Nutritional Intervention

After a six-month follow-up period, the effect of adherence on patient outcomes in the low-adherence group showed a significant decrease in anthropometrics (weight and BMI), BIVA (PA, SPA, BCM, BCMI, FFM, FFMI, SMM, ASMM, SMI, MM), US muscle mass quantity (RF-CSA, RF-CIRC, RF-*X*-axis, RF-*Y*-axis) and functional assessment (handgrip strength) Vs baseline (significance in Supplementary Table S3). However, in the high- adherence group there was an increase in anthropometrics (weight and BMI), BIVA, US muscle mass quantity and functional assessment (handgrip strength) Vs baseline. Significance is described in Table 3. 

**Figure 2:**
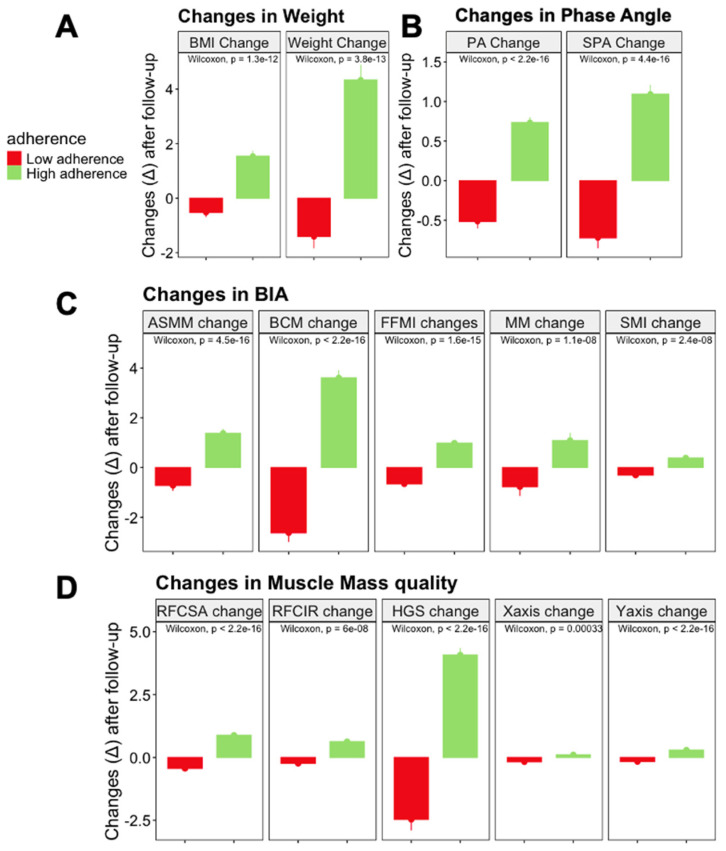
Changes in morphofunctional and anthropometric parameters by adherence group. Panel (**A**) shows the increase in body weight (kg) after six months of nutritional intervention, comparing high-adherence and low-adherence groups. Panel (**B**) illustrates improvements in phase angle (PA,°), reflecting enhanced cellular integrity in both groups, with greater gains in the high-adherence group. Panel (**C**) highlights changes in BIA and Panel (**D**) illustrates rectus femoris cross-sectional area (RF-CSA, cm^2^) and handgrip strength (HGS, kg), demonstrating superior muscle mass recovery and functional improvement in the high-adherence group. Significant differences between the means of the different groups of subjects was performed according to the Wilcoxon test (*p* < 0.05). **Abbreviations**: PA, phase angle; RF-CSA, rectus femoris cross-sectional area; HGS, handgrip strength.

When focusing on the rate of change between these groups, we observed that the high-adherence group showed greater increases in weight and BMI compared to the low- adherence group (both *p* < 0.001) (Figure 2A). Regarding BIVA variables, the high-adherence group exhibited greater changes in PA and SPA compared to the low-adherence group (both *p* < 0.001) (Figure 2B). In terms of muscle quantity and quality, measured by BIVA the high-adherence group demonstrated larger changes in ASMM, FFMI, SMI, and other parameters related to nutritional ultrasound, such as RF-CSA, RF-CIRC, and the *X*- and *Y*-axis, when compared to the low-adherence group (all *p* < 0.001) (Figure 2C,D) (Supplementary Figure S2A). To test whether baseline nutritional variables assessed in this study were the most valuable for predicting overall survival, we used a Random Forest analysis to determine this analysis. The Random Forest test showed that handgrip strength and RF-CSA were the most important variables used to predict mortality in our analysis (Supplementary Figure S2B). 

### 3.6. GLIM and Sarcopenia Recovery After Nutritional Intervention According to the Adherence to Nutritional Supplement

**Figure 3:**
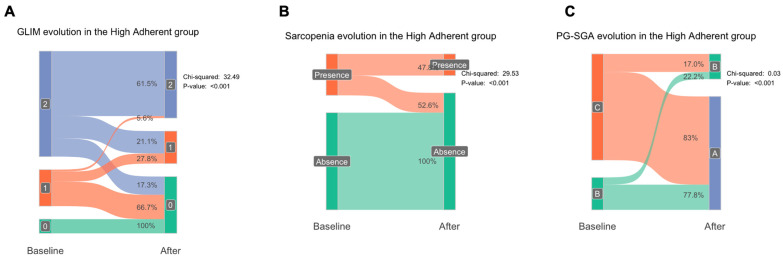
Diagnostic and functional recovery in high-adherence group. This figure illustrates the improvements observed in the high-adherence group after six months of nutritional intervention. Panel (**A**) shows significant changes in the Global Leadership Initiative on Malnutrition (GLIM) criteria, with a higher percentage of patients improving from severe (criteria 2) to moderate (criteria 1) or no malnutrition (criteria 0). Panel (**B**) highlights shifts in Subjective Global Assessment (SGA) categories, reflecting substantial recovery from severe malnutrition (C to A or B). Panel (**C**) depicts sarcopenia recovery rates, showing that the majority of sarcopenic patients achieved functional and nutritional recovery. Chi-Square test was conducted and indicated significant difference between baseline and after nutritional intervention. **Abbreviations:** GLIM, Global Leadership Initiative on Malnutrition; SGA, Subjective Global Assessment.

To understand the effect of the nutritional intervention on the GLIM, PS-SGA, and sarcopenia criteria, we assessed these parameters at baseline and after a six-month follow-up period. We observed that, among patients with high adherence, 21.1% of those classified under GLIM criteria 2 (Severe malnutrition) moved to criteria 1 (Moderate malnutrition), and 17.3% improved to criteria 0 (No malnutrition). In contrast, only 2.4% of patients with GLIM criteria 2 in the low-adherence group moved to criteria 1, and none improved to criteria 0 (Figure 3, Supplementary Figure S3). Among patients in criteria 1, 66.7% returned to criteria 0, while 27.8% remained in criteria 1 in the high-adherence group, compared to 33.3% of patients in the low-adherence group who returned to criteria 0. For those initially classified under criteria 0, 100% remained in criteria 0 in the high-adherence group, while 50% of patients in the low-adherence group moved to criteria 1 and 50% maintained criteria 0 status (*p* < 0.001). Regarding the PG-SGA, 77.8% of patients in criteria B improved to criteria A, and 83% of patients in criteria C moved to criteria A in the high- adherence group. However, none of the patients in criteria B in the low-adherence group improved to criteria A, and only 2.1% of patients in criteria C moved to criteria A (*p* < 0.001). For sarcopenia, 52.7% of sarcopenic patients recovered in the high-adherence group, compared to only 5.9% of sarcopenic patients in the low-adherence group (*p* < 0.001).

### 3.7. Changes in Nutritional Assessment Variables on Mortality

**Figure 4:**
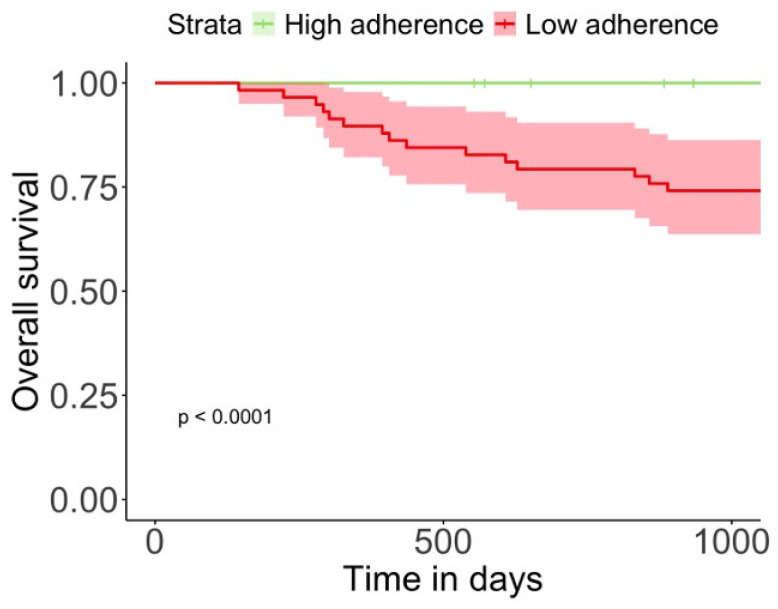
Kaplan–Meier survival curves by adherence group. This figure presents Kaplan–Meier survival curves comparing the high-adherence and low-adherence groups over a 24-month period. The high-adherence group demonstrates significantly better survival rates compared to the low-adherence group (*p* < 0.001). Survival differences emphasize the prognostic importance of adherence to the nutritional intervention.

We performed a Kaplan–Meier analysis, comparing the overall survival of two groups: high adherence and low adherence. The analysis revealed that the low-adherence group had a significantly lower overall survival rate compared to the high-adherence group (*p* < 0.001) (Figure 4). Finally, to understand how these changes in BIVA, muscle mass, and functional assessment improved the survival, we conducted a multivariate Cox analysis (Table 4). The change in PA showed a strong association with reduced mortality, with an HR of 0.27 (95% CI: 0.15–0.50, *p* < 0.001). The optimal cut-off point for PA changes was identified at 0.1°, achieving a sensitivity of 67.5% and a specificity of 93.3%, with an AUC of 0.848. Changes in SPA also indicated a significant mortality risk reduction, with an HR of 0.39 (95% CI: 0.25–0.59, *p* < 0.001). The cut-off for SPA changes was −0.1, yielding a sensitivity of 69.1% and a specificity of 93.3%, with an AUC of 0.844. An increase in BCM changes was significantly associated with reduced mortality, with an HR of 0.74 (95% CI: 0.66–0.84, *p* < 0.001). The cut-off for BCM changes was −0.2 kg, providing a sensitivity of 71.7% and perfect specificity (100%), with an AUC of 0.885. Changes in BCMI were similarly predictive, with an HR of 0.38 (95% CI: 0.26–0.57, *p* < 0.001). The cut-off point was −0.2, with a sensitivity of 75.0% and a specificity of 93.3%, resulting in an AUC of 0.871. FFM changes increases were associated with reduced mortality risk, indicated by an HR of 0.71 (95% CI: 0.58–0.87, *p* < 0.001). A cut-off of −1.6 kg was established, with a high sensitivity of 80.8% and specificity of 73.3%, and an AUC of 0.765. Similarly, changes in FFMI had an HR of 0.34 (95% CI: 0.19–0.62, *p* < 0.001), with a cut-off of −0.5, yielding a sensitivity of 79.2% and specificity of 80%, with an AUC of 0.760. As a functional parameter, handgrip strength showed a significant association with mortality, with an HR of 0.82 (95% CI: 0.75–0.89, *p* < 0.001). A cut-off of −1.0 kg resulted in a sensitivity of 75.0% and perfect specificity (100%), with an AUC of 0.923. Conversely, changes in fat mass (FM), skeletal muscle index (SMI), and total body water (TBW) did not show significant associations with mortality, with *p*-values of 0.379, 0.345, and 0.132.

## 4. Discussion

The findings of this study provide compelling evidence for the significant impact of adherence to a nutritional supplement on various health outcomes in a population at risk of malnutrition and sarcopenia. The observed association between high adherence and improved survival, as well as the marked differences in morphofunctional parameters between the high- and low-adherence groups, underscore the critical role of nutritional interventions in this vulnerable population. We observed that nutritional interventions improved the majority of body measurements. However, the major impact was observed in the high-adherence group, in which they improved their body measurement significantly when compared to the low-adherence group, as well as having better survival rates than the low-adherence group. This study underscores the relevance of the adherence of the nutritional supplements in malnourished patients.

The phase angle is a recognized marker for disease-related malnutrition, with reductions in its value associated with increased mortality and poorer metabolic, nutritional, and disease progression profiles in malnourished patients. This measurement reflects cell membrane integrity and muscular function, which are impacted by cellular health [19]. In our study, however, a statistically significant improvement in phase angle was observed with nutritional supplementation, indicating enhanced cellular integrity and potential functional recovery. Our findings align with those of Cornejo et al., who reported a significant phase angle (PA) increase of 0.8° (from 5.2° to 6.0°, *p* < 0.05) in patients receiving an HMB-enriched supplement, reflecting improved cellular integrity and muscle quantity and quality [17]. Similarly, our high-adherence group showed a PA increase from 4.78° to 5.52° (*p* < 0.001), while the low-adherence group experienced a decline (from 5.14° to 4.62°, *p* < 0.001), suggesting weakened cellular health due to insufficient supplementation. 

Handgrip strength (HGS) serves as a valuable indicator of nutritional status and functional recovery, with evidence from the review by Sanz-Paris et al. showing that HMB-enriched nutritional supplements can improve muscle function, particularly in malnourished, sarcopenic patients [14]. The review highlights that significant HGS gains, particularly in patients with notable weight recovery, correlate with improved clinical outcomes, underscoring the potential role of HGS in evaluating the efficacy of nutritional interventions. Additionally, Cornejo et al. observed a 6.9 kg improvement in handgrip strength, consistent with our 4.1 kg increase in the high-adherence group, highlighting the critical impact of adherence to HMB supplementation on functional and cellular health in malnourished patients (15).

The RF-CSA is a significant prognostic indicator of mortality in cancer patients, as lower RF-CSA values are independently associated with higher mortality risk, underscoring its clinical relevance for nutritional assessment and prediction of outcomes in disease-related malnutrition [20]. The increase in RF-CSA observed in our study underscores the significance of nutritional intervention for muscle mass recovery in malnourished patients. Improvements in RF-CSA are particularly relevant, as the rectus femoris muscle is a critical predictor of functional strength and mobility, factors directly impacting quality of life and clinical prognosis. By comparison, the study by Lattanzi et al. assessed quadriceps muscle thickness via ultrasound in patients with liver cirrhosis, similarly reporting a significant increase in the HMB-supplemented group compared to controls. Although Lattanzi et al. measured thickness rather than RF-CSA, both studies highlight HMB’s potential to counteract muscle wasting and support functional recovery in vulnerable populations [21].

While the exact mechanisms underlying the beneficial effects of the nutritional supplement remain to be fully elucidated, several plausible explanations can be proposed. The supplement may have acted by providing essential nutrients, and helped to correct nutrient deficiencies and improve overall nutritional status, leading to enhanced tissue repair and function. Chronic inflammation is associated with aging, malnutrition, and sarcopenia. The supplement may have exerted anti-inflammatory effects, thereby mitigating the negative consequences of inflammation on muscle mass and function.

Adherence to nutritional interventions is often suboptimal, and several factors may have contributed to the observed differences in adherence. In our study, these factors—age, education level, socioeconomic status and comorbidities—were controlled for, and no significant differences were found between groups. This suggests that the observed differences in adherence were not attributable to these variables. Patients’ beliefs about the effectiveness of the intervention, as well as their motivation and self-efficacy, may have influenced their adherence behavior.

The article by Wan et al. (2021) focuses on factors influencing adherence to oral nutritional supplementation (ONS) in patients’ post-gastric cancer surgery, identifying key barriers and facilitators [22]. The study protocol outlines various determinants, such as patient age, socioeconomic status, education level, and comorbidities, which may impact patients’ ability to adhere to prescribed nutritional regimens. Additionally, factors like patients’ beliefs about the efficacy of supplementation, motivation, and self-efficacy are considered as potential influences on adherence behavior. This study offers valuable insights into the complexity of adherence, highlighting the need to address these determinants to improve clinical outcomes in nutritional interventions [22]. Additionally, our findings build upon previous evidence, such as the randomized controlled trial by Deutz et al. (2016) [12], which demonstrated functional improvements over 12 weeks of HMB-ONS intervention. However, our study extends these observations by providing a six-month follow-up in a real-world clinical setting, capturing adherence levels and their impact on both morphofunctional markers and survival. Notably, we report for the first time a strong association between changes in phase angle and handgrip strength with medium-term mortality reduction, underscoring the prognostic value of these indicators and supporting the relevance of adherence-monitoring strategies in routine care.

Future research should focus on the following areas: Longer-term follow-up studies are needed to assess the sustainability of the observed benefits. Further investigations are required to elucidate the specific mechanisms by which the nutritional supplement exerts its effects. Cost-effectiveness analyses are needed to evaluate the economic implications of implementing nutritional interventions. The development of personalized nutritional plans based on individual genetic and metabolic profiles may improve outcomes. This study has several limitations that should be considered when interpreting the results. These include the following: The observational design of the study limits the ability to establish causality. The results may not be generalizable to other populations or settings. There may be unmeasured confounding factors that influenced the outcomes. Additionally, this retrospective observational study was designed to capture the real-world effectiveness of a six-month multimodal nutritional intervention in malnourished outpatients from various clinical backgrounds. While randomized controlled trials (RCTs) provide high internal validity, observational designs offer important insights into clinical implementation across heterogeneous populations. Our study complements prior RCTs by demonstrating consistent improvements in morphofunctional parameters and survival in a broader clinical setting. The inclusion of real-life adherence data and the association with outcomes enhances the translational value of our findings. Moreover, the diverse diagnoses and outpatient nature of the cohort reflect the complexity of malnutrition management in daily clinical practice.

## 5. Conclusions

In conclusion, this study suggests that a structured nutritional recovery program with HMB-enriched oral supplements, combined with dietary recommendations and adapted exercise, significantly improves survival and key morphofunctional parameters in malnourished patients. Notable improvements were observed in phase angle, rectus femoris cross-sectional area, handgrip strength, and functional mobility, reflecting enhanced nutritional and functional recovery. These benefits were most pronounced in patients with high adherence, highlighting the critical role of compliance in achieving optimal outcomes. Furthermore, the observed association between changes in morphofunctional markers and reduced mortality underscores their prognostic value. These findings support the implementation of comprehensive nutritional support programs in clinical practice and emphasize the need for further research to optimize long-term strategies.

## Figures and Tables

**Table 1:** Baseline characteristics of the population included in the study divided by sex.

	All	Women	Men	*p* Value
Variables	N = 135	N = 75	N = 60	
**Anthropometric and demographic variables**				
Age, years	61.3 (14.7)	62.2 (12.9)	60.2 (16.8)	0.672
Sex:				-
Female	75 (55.6%)	-	-	
Male	60 (44.4%)	-	-	
Body-mass index, kg/m^2^	22.0 (3.83)	21.7 (4.04)	22.2 (3.57)	0.307
**BIVA variables**				
PA, (°)	4.93 (1.02)	4.71 (0.90)	5.22 (1.09)	0.004 **
SPA	−0.22 (1.49)	0.24 (1.47)	−0.79 (1.30)	<0.001 ***
BCM, kg	22.3 (6.29)	18.9 (3.63)	26.5 (6.43)	<0.001 ***
BCMI, kg/m^2^	8.10 (1.83)	7.41 (1.36)	8.95 (1.99)	<0.001 ***
FFM, kg	46.6 (8.92)	41.0 (4.64)	53.7 (7.91)	<0.001 ***
FFMI, kg/m^2^	17.0 (2.17)	16.1 (1.65)	18.2 (2.19)	<0.001 ***
FM, kg	13.4 (7.02)	14.4 (7.48)	12.0 (6.20)	0.106
FMI, kg/m^2^	4.95 (2.70)	5.66 (2.91)	4.07 (2.12)	0.002 **
SMM, kg	22.3 (6.38)	17.7 (3.30)	28.0 (4.32)	<0.001 ***
ASMM, kg	17.2 (4.35)	14.3 (2.41)	20.7 (3.61)	<0.001 ***
SMI, kg/m^2^	8.06 (1.74)	6.92 (1.18)	9.49 (1.18)	<0.001 ***
MM, kg	22.3 (6.38)	17.7 (3.30)	28.0 (4.32)	<0.001 ***
ECW, kg	17.8 (3.43)	16.0 (2.51)	20.0 (3.16)	<0.001 ***
TBW, kg	34.6 (6.94)	30.3 (3.98)	40.0 (6.01)	<0.001 ***
Na/K	1.29 (0.26)	1.27 (0.27)	1.31 (0.25)	0.167
Hydragram^®^, %	73.9 (2.91)	73.6 (3.11)	74.2 (2.61)	0.189
Nutrigram^®^, mg/24 h/m	671 (184)	569 (104)	798 (183)	<0.001 ***
**Nutritional ultrasound**				
RF-CSA, cm^2^	3.17 (1.26)	2.64 (0.84)	3.86 (1.39)	<0.001 ***
RF-CIRC, cm	8.31 (1.39)	7.69 (1.13)	9.08 (1.29)	<0.001 ***
RF-*X*-axis, cm	3.58 (0.61)	3.32 (0.54)	3.90 (0.54)	<0.001 ***
RF-*Y*-axis, cm	1.02 (0.31)	0.93 (0.26)	1.13 (0.33)	0.001 **
L-SAT, cm	0.75 (0.46)	0.98 (0.46)	0.46 (0.25)	<0.001 ***
T-SAT, cm	1.32 (0.58)	1.36 (0.69)	1.26 (0.40)	0.869
S-SAT, cm	0.62 (0.31)	0.68 (0.37)	0.55 (0.20)	0.109
VAT, cm	0.37 (0.20)	0.37 (0.18)	0.38 (0.23)	0.834
**Functional parameters**				
Handgrip strength, kg	24.3 (9.73)	19.4 (5.72)	30.4 (10.2)	<0.001 ***
Up and Go, s	7.54 (1.97)	7.75 (2.07)	7.29 (1.83)	0.150

Data are expressed as mean ± standard deviation (SD) for continuous variables and as number and percentage [n (%)] for categorical variables. ** *p* < 0.01, *** *p* < 0.001. **Abbreviations**: BCM: body cell mass; BCMI: BCM index; BMI: body mass index; BIVA: bioelectrical impedance vectorial analysis; FM: fat mass; FMI: FM index; FFMI: fat-free mass index; PA: phase angle; RF-CIR: circumference of quadriceps rectus femoris; RF-CSA: rectus femoris cross-sectional area; SAT: subcutaneous adipose fat of leg (L), superficial (S) and total (T) abdominal; SMI: skeletal muscle index; SPA: standardized phase angle.

**Table 2:** Changes in nutritional status after nutritional intervention in the patients included in the study.

Variable	Baseline Mean	SD	After Mean	SD	*p* Value	Change	Difference
**Anthropometric data**							
Weight, kg	59.976	12.233	61.844	13.396	0.000 ***	3.139	1.868
BMI, kg/m^2^	21.950	3.834	22.605	4.260	0.000 ***	3.051	0.655
**BIVA variables**							
PA, º	4.935	1.016	5.133	1.116	0.010 *	5.346	0.198
SPA	−0.219	1.487	0.095	1.565	0.008 **		0.314
BCM, kg	22.291	6.294	23.222	6.877	0.010 *	5.514	0.931
BCMI, kg/m^2^	8.098	1.830	8.421	2.034	0.009 **	5.260	0.324
FFM, kg	46.616	8.917	47.453	9.509	0.009 **	1.907	0.837
FFMI, kg/m^2^	16.997	2.166	17.278	2.383	0.013 *	1.803	0.281
FM, kg	13.361	7.022	14.392	7.912	0.004 **	14.426	1.031
FMI, kg/m^2^	4.953	2.700	5.321	3.014	0.004 **	14.318	0.369
SMM, kg	22.281	6.381	22.570	6.713	0.247	1.806	0.290
ASMM, kg	17.161	4.353	17.639	4.623	0.004 **	3.122	0.478
SMI, kg/m^2^	8.059	1.738	8.149	1.836	0.317	1.665	0.090
MM, kg	22.281	6.381	22.570	6.713	0.247	1.806	0.290
ECW, kg	17.780	3.433	17.773	4.165	0.976	0.078	−0.007
TBW, kg	34.583	6.943	35.188	7.355	0.023 *	1.938	0.605
Na/K	1.287	0.259	1.264	0.419	0.468	−1.218	−0.023
Hydragram^®^, %	73.877	2.906	74.042	3.276	0.520	0.279	0.165
Nutrigram^®^, mg/24 h/m	670.737	183.734	698.041	201.236	0.010 *	5.333	27.304
**Nutritional ultrasound**							
RF-CSA, cm^2^	3.174	1.264	3.498	1.513	0.000 **	12.071	0.323
RF-CIRC, cm	8.306	1.386	8.571	1.461	0.001 ***	3.631	0.265
RF-*X*-axis, cm	3.578	0.611	3.564	0.610	0.724	0.450	−0.014
RF-*Y*-axis, cm	1.017	0.311	1.114	0.370	0.000 ***	12.995	0.097
L-SAT, cm	0.751	0.459	0.809	0.513	0.007 **	11.772	0.058
T-SAT, cm	1.317	0.583	1.411	0.796	0.131	9.802	0.094
S-SAT, cm	0.621	0.311	0.664	0.450	0.105	12.737	0.043
VAT, cm	0.372	0.200	0.427	0.286	0.678	22.931	0.056
**Functional parameters**							
Handgrip, kg	24.296	9.727	25.563	9.971	0.001 **	6.845	1.267
Up and Go, s	7.543	1.969	7.074	1.863	0.001 **	−4.715	−0.468

Data are expressed as mean ± standard deviations. A Shapiro–Wilks test was performed to decide between normal or non-normal. Asterisk indicates significant differences between groups according to the paired *t*-test or Wilcoxon test according to the normality of the variables (*** *p* < 0.001, ** *p* < 0.01, * *p* < 0.05). **Abbreviations**: BCM: body cell mass; BCMI: BCM index; BMI: body mass index; BIVA: bioelectrical impedance vectorial analysis; FM: fat mass; FMI: FM index; FFMI: fat-free mass index; PA: phase angle; RF-CIR: circumference of quadriceps rectus femoris; RF-CSA: rectus femoris cross-sectional area; SAT: subcutaneous adipose fat of leg (L), superficial (S) and total (T) abdominal; SMI: skeletal muscle index; SPA: standardized phase angle.

**Table 3:** Changes in nutritional status after nutritional intervention in the high-adherent group.

Variable	Baseline Mean	SD	After Mean	SD	*p* Value	Change (%)	Difference
**Anthropometric variables**							
Weight, kg	61.283	11.398	65.623	12.254	0.000 ***	7.338	4.340
Body-mass index, kg/m^2^	22.378	3.639	23.922	3.916	0.000 ***	7.164	1.544
**BIA variable**							
PA, °	4.781	0.952	5.516	1.028	0.000 ***	16.513	0.735
SPA	−0.557	1.353	0.538	1.343	0.000 ***	NA	1.095
BCM, kg	21.921	6.330	25.527	6.959	0.000 ***	17.685	3.606
BCMI, kg/m^2^	7.951	1.809	9.188	1.918	0.000 ***	16.840	1.238
FFM, kg	46.797	8.979	49.603	9.688	0.000 ***	6.095	2.805
FFMI, kg/m^2^	17.003	2.168	17.988	2.399	0.000 ***	5.900	0.986
FM, kg	14.486	7.050	16.021	7.727	0.005 **	21.539	1.535
FMI, kg/m^2^	5.375	2.746	5.930	2.993	0.005 **	21.476	0.555
SMM, kg	22.344	6.663	23.434	6.691	0.001 **	5.897	1.090
ASMM, kg	17.257	4.440	18.636	4.558	0.000 ***	8.645	1.379
SMI, kg/m^2^	8.051	1.831	8.439	1.871	0.001 **	5.646	0.388
MM, kg	22.344	6.663	23.434	6.691	0.001 **	5.897	1.090
ECW, kg	18.113	3.430	17.536	3.189	0.006 **	−2.646	−0.577
TBW, kg	34.700	7.121	36.475	7.336	0.000 ***	5.447	1.775
Na/K	1.284	0.242	1.139	0.231	0.000 ***	−10.650	−0.145
Hydragram^®^, %	73.913	3.039	73.469	2.241	0.132	−0.511	−0.444
Nutrigram^®^, mg/24 h/m	660.483	183.749	764.932	202.153	0.000 ***	16.981	104.449
**Nutritional ultrasound**							
RF-CSA, cm^2^	3.088	1.262	3.986	1.584	0.000 ***	30.836	0.899
RF-CIRC, cm	8.199	1.510	8.845	1.532	0.000 ***	8.472	0.647
RF-*X*-axis, cm	3.525	0.677	3.631	0.655	0.058	4.183	0.106
RF-*Y*-axis, cm	0.991	0.276	1.286	0.338	0.000 ***	33.086	0.295
L-SAT, cm	0.755	0.434	0.876	0.506	0.000 ***	21.096	0.122
T-SAT, cm	1.383	0.605	1.598	0.813	0.010 *	22.780	0.215
S-SAT, cm	0.636	0.316	0.750	0.465	0.020 *	22.014	0.115
VAT, cm	0.369	0.218	0.431	0.245	0.207	45.614	0.062
**Functional parameters**							
Handgrip, kg	23.532	8.729	27.610	9.687	0.000 ***	18.800	4.078
Up and Go, s	7.942	2.210	6.489	1.743	0.000 ***	−17.649	−1.454

Data are expressed as mean ± standard deviations. A Shapiro–Wilks test was performed to decide between normal or non-normal. Asterisk indicates significant differences between groups according to the paired *t*-test or Wilcoxon test according to the normality of the variables (*** *p* < 0.001, ** *p* < 0.01, * *p* < 0.05). **Abbreviations**: BCM: body cell mass; BCMI: BCM index; BMI: body mass index; BIVA: bioelectrical impedance vectorial analysis; FM: fat mass; FMI: FM index; FFMI: fat-free mass index; PA: phase angle; RF-CIR: circumference of quadriceps rectus femoris; RF-CSA: rectus femoris cross-sectional area; SAT: subcutaneous adipose fat of leg (L), superficial (S) and total (T) abdominal; SMI: skeletal muscle index; SPA: standardized phase angle.

**Table 4:** Changes in nutritional assessment after nutritional intervention and the mortality risk.

Variables	HR (CI 95%)	Cut-Ooff Point	Sensitivity *(%)*	Specificity (%)	AUC	*p* Value
**Changes in BIVA variables**						
PA, (º)	0.27 (0.15–0.50)	0.1	0.675	0.933	0.848	<0.001 ***
SPA	0.39 (0.25–0.59)	−0.1	0.691	0.933	0.844	<0.001 ***
BCM, kg	0.74 (0.66–0.84)	−0.2	0.717	1.00	0.885	<0.001 ***
BCMI, kg/m^2^	0.38 (0.26–0.57)	−0.2	0.750	0.933	0.871	<0.001 ***
FFM, kg	0.71 (0.58–0.87)	−1.6	0.808	0.7333	0.765	<0.001 ***
FFMI, kg/m^2^	0.34 (0.19–0.62)	−0.5	0.792	0.800	0.760	<0.001 ***
FM, kg	0.94 (0.82–1.08)	0.2	0.617	0.667	0.576	0.379
FMI, kg/m^2^	0.85 (0.58–1.25)	0.1	0.617	0.667	0.584	0.413
SMI, kg/m^2^	0.72 (0.37–1.42)	−0.2	0.685	0.733	0.663	0.345
ECW, kg	1.15 (1.05–1.26)	0.4	0.733	0.675	0.677	0.001 **
TBW, kg	0.85 (0.69–1.05)	−0.9	0.733	0.733	0.720	0.132
Na/K	2.68 (1.59–4.50)	0.0	0.933	0.592	0.789	<0.001 ***
Hydragram^®^, %	1.24 (1.12–1.36)	0.2	0.733	0.717	0.721	<0.001 ***
Nutrigram^®^, mg/24 h/m	0.99 (0.98–0.99)	−14.6	0.742	0.933	0.873	<0.001 ***
**Changes in Nutritional ultrasound**						
RF-CSA, cm^2^	0.36 (0.19–0.69)	−0.07	0.731	0.867	0.792	0.002 **
RF-CIRC, cm	0.70 (0.36–1.34)	1.21	0.176	1.000	0.547	0.280
RF-*X*-axis, cm	0.61 (0.17–2.12)	0.09	0.600	0.592	0.500	0.433
RF-*Y*-axis, cm	0.04 (0.00–0.25)	0.02	0.692	1.00	0.835	<0.001 ***
L-SAT, cm	0.08 (0.01–0.59)	0.11	0.500	1.000	0.681	0.013 *
T-SAT, cm	0.43 (0.13–1.42)	0.13	0.409	0.833	0.595	0.166
S-SAT, cm	0.02 (0.00–0.38)	0.00	0.568	0.917	0.703	0.010 *
VAT, cm	0.37 (0.03–4.73)	0.02	0.500	1.000	0.680	0.448
**Changes in Functional parameters**						
Handgrip strength, kg	0.82 (0.75–0.89)	−1.0	0.750	1.000	0.923	<0.001 ***
Up and Go, s	6.37 (2.43–16.73)	1.1	1.000	0.858	0.934	<0.001 ***

The table presents results on various body composition and functional parameters to determine whether changes in these variables are predictors of mortality. Hazard ratios (HR) with 95% confidence intervals (CI) adjusted by age, sex, and BMI, cut-off points, sensitivity, specificity, area under the ROC curve (AUC), and *p*-values are shown for each variable. Significant predictors of reduced mortality are highlighted with symbols indicating different levels of statistical significance (*** *p* < 0.001, ** *p* < 0.01, * *p* < 0.05). **Abbreviations**: BCM: body cell mass; BCMI: BCM index; BMI: body mass index; BIVA: bioelectrical impedance vectorial analysis; FM: fat mass; FMI: FM index; FFMI: fat-free mass index; PA: phase angle; RF-CIR: circumference of quadriceps rectus femoris; RF-CSA: rectus femoris cross-sectional area; SAT: subcutaneous adipose fat of leg (L), superficial (S) and total (T) abdominal; SMI: skeletal muscle index; SPA: standardized phase angle.

## Data Availability

The data that support the findings of this study are available on request from the corresponding author. The data are not publicly available due to privacy or ethical restrictions.

## Supplementary Materials

The following supporting information can be downloaded at: https://www.mdpi.com/article/10.3390/nu17091601/s1, Supplementary Figure S1: Flow chart of the patients included in the study. Flowchart illustrating the selection process from the initial cohort of 148 patients to the final analyzed population of 135 patients. Reasons for exclusion included lack of adherence to the intervention, incomplete data for bioelectrical impedance analysis (BIVA), and missing follow-up evaluations. Supplementary Figure S2: Changes in bioelectrical impedance analysis (BIVA) parameters and feature importance. Panel A shows the changes (Δ) in key BIVA parameters after follow-up, comparing high-adherence and low-adherence groups. Improvements in parameters such as appendicular skeletal muscle mass (ASMM), body cell mass (BCM), and fat-free mass index (FFMI) are significantly greater in the high-adherence group (Wilcoxon, *p* < 0.001). Panel B presents the variable importance plot (VIP) from the analysis, highlighting handgrip strength (HGS), rectus femoris cross-sectional area (RF-CSA), and skeletal muscle index (SMI) as the most predictive factors influencing outcomes. Abbreviations: BIVA, bioelectrical impedance analysis; ASMM, appendicular skeletal muscle mass; BCM, body cell mass; FFMI, fat-free mass index; HGS, handgrip strength; RF-CSA, rectus femoris cross-sectional area; SMI, skeletal muscle index; SPA, standardized phase angle; TBW, total body water. Supplementary Figure S3: Diagnostic and functional changes in low-adherence group. This figure illustrates the changes observed exclusively in the low-adherence group after six months of nutritional intervention. Panel A shows shifts in the Global Leadership Initiative on Malnutrition (GLIM) criteria, with limited improvements compared to the high-adherence group. Panel B presents changes in Subjective Global Assessment (SGA) categories, showing modest recovery rates from severe malnutrition. Panel C highlights sarcopenia recovery rates, which are notably lower in this group compared to high-adherence patients. Chi-Square test was conducted to indicate significant difference between baseline and after nutritional intervention (*** *p* < 0.001, ** *p* < 0.01, * *p* < 0.05). Supplementary Figure S4: Ultrasound images of rectus-femoris and abdominal muscle assessment.
