# Supplementary material for: Adherence to β-hydroxy-β-methylbutyrate-Enriched Oral Nutritional Supplements Enhances Survival and Nutritional Recovery in Malnourished Outpatients: Prognostic Insights
Source: Nutrients. 2025 May 7;17(9):1601. doi: 10.3390/nu17091601 (PMC12073151; doi:10.3390/nu17091601)
Supplement: Supplementary file 1 [file nutrients-17-01601-s001.zip › Supplementary Figure S1.pptx]

## Slide 1
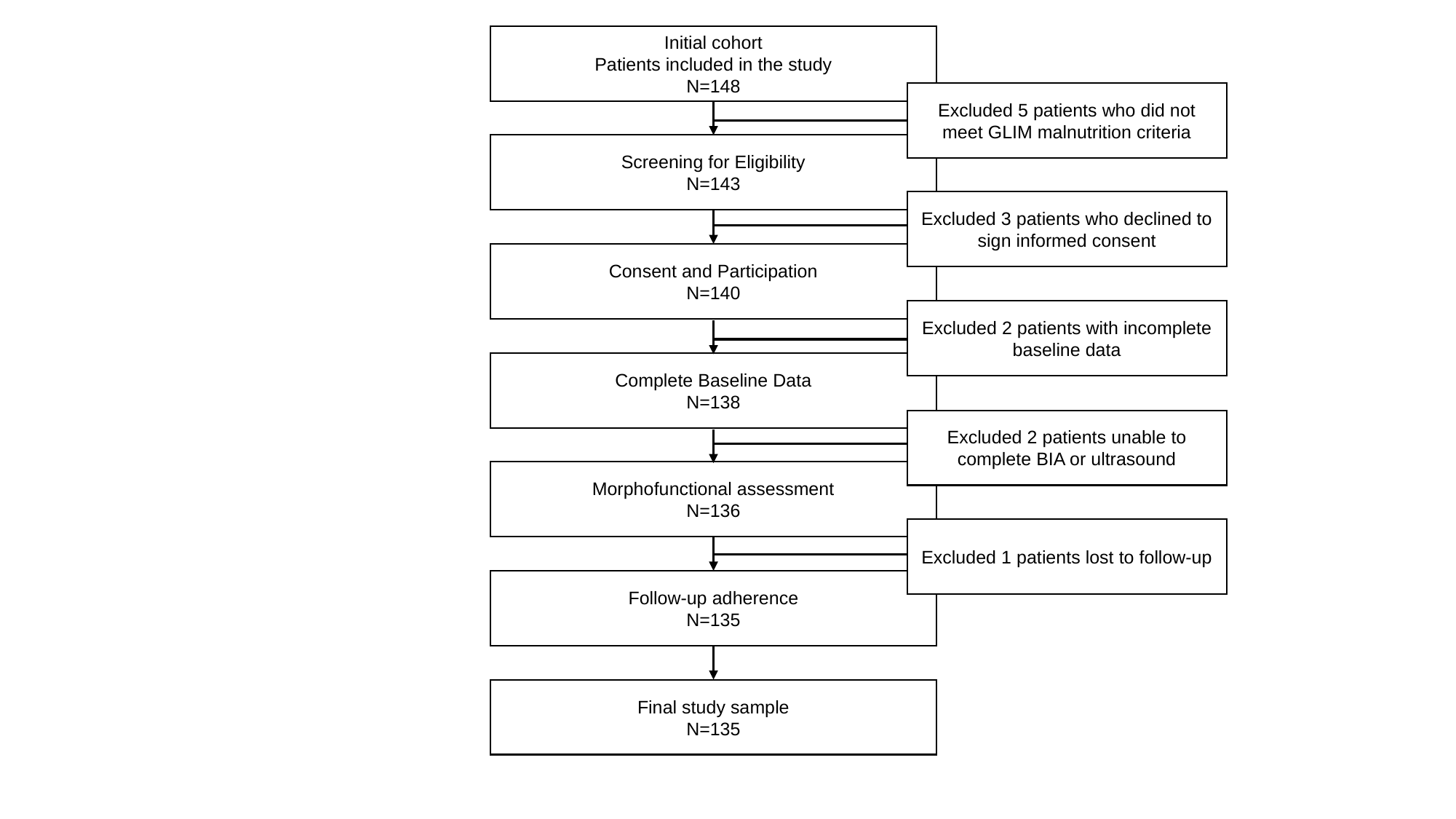

Initial cohort
Patients included in the study
N=148
Excluded 5 patients who did not meet GLIM malnutrition criteria
Screening for Eligibility
N=143
Excluded 3 patients who declined to sign informed consent
Consent and Participation
N=140
Excluded 2 patients with incomplete baseline data
Complete Baseline Data
N=138
Excluded 2 patients unable to complete BIA or ultrasound
Morphofunctional assessment
N=136
Excluded 1 patients lost to follow-up
Follow-up adherence
N=135
Final study sample
N=135
