# Supplementary material for: Adherence to β-hydroxy-β-methylbutyrate-Enriched Oral Nutritional Supplements Enhances Survival and Nutritional Recovery in Malnourished Outpatients: Prognostic Insights
Source: Nutrients. 2025 May 7;17(9):1601. doi: 10.3390/nu17091601 (PMC12073151; doi:10.3390/nu17091601)
Supplement: Supplementary file 1 [file nutrients-17-01601-s001.zip › Supplementary Table S1.pdf]

Supplementary Table S1. Grouping of Pathologies by Category and disease type

Type	Subtype	Percentage (%)
Cancer (N=94)		69.7
	Colon cancer	3.3
	Rectal cancer	4.4
	Gastric cancer	21.3
	Esophageal cancer	3.3
	Head and neck cancer	3.3
	Gastrointestinal estromal tumor	2.2
	Breast cancer	2.2
	Ovarian cancer	2.2
	Prostate cancer	3.3
	Renal Leiomyosarcoma	1.1
	Ewing Sarcoma	1.1
	Micro-sarcoma	1.1
	Lymphoma	1.1
Inflammatory Bowel disease (N=11)		7.8
	Inflammatory Bowel Disease	4.5
	Crohn's Disease	2.2
	Lymphocytic Colitis	1.1
Digestive System Diseases (N=31)		23.5
	Pancreatitis	6.7
	Jejunal Resection	1.1
	Esophageal Atresia	1.1
	Esophagectomy	1.1
	Cephalic duodenopancreatectomy	10.1
	Exocrine Pancreatic Insufficiency	3.4
Neuromuscular and Autoimmune Diseases (N=6)		4.4
	Lupus	1.1
	Mitochondrial Myopathy	1.1
	Polyneuropathy	1.1
	Scleroderma	1.1
Other diseases (N=6)		4.4
	Adrenal Insufficiency	1.1
	Appendicular Plastron	1.1
	Stroke	1.1
	Pulmonary Fibrosis	1.1
