# Supplementary material for: Adherence to β-hydroxy-β-methylbutyrate-Enriched Oral Nutritional Supplements Enhances Survival and Nutritional Recovery in Malnourished Outpatients: Prognostic Insights
Source: Nutrients. 2025 May 7;17(9):1601. doi: 10.3390/nu17091601 (PMC12073151; doi:10.3390/nu17091601)
Supplement: Supplementary file 1 [file nutrients-17-01601-s001.zip › Supplementary Table S2.pdf]

Supplementary Table S2. Baseline characteristics of the population of study divided by Nutritional Adherence

	**Low adherence** *N=58*	**High adherence** *N=77*	***P* value**	**N**
**Anthropometric and demographic variables**				
Age, years	62.9 (13.5)	60.0 (15.6)	0.325	135
Sex:			0.923	135
Female	33 (56.9%)	42 (54.5%)		
Male	25 (43.1%)	35 (45.5%)		
Body-mass index, kg/m²	21.4 (4.04)	22.4 (3.64)	0.116	135
**BIVA**				
PA, (º)	5.14 (1.07)	4.78 (0.95)	0.045*	135
SPA	0.23 (1.55)	-0.56 (1.35)	0.003**	135
BCM, kg	22.8 (6.27)	21.9 (6.33)	0.338	135
BCMI, kg/m^2	8.29 (1.86)	7.95 (1.81)	0.187	135
FFM, kg	46.4 (8.91)	46.8 (8.98)	0.783	135
FFMI, kg/m^2	17.0 (2.18)	17.0 (2.17)	0.984	135
FM, kg	11.9 (6.76)	14.5 (7.05)	0.023*	135
FMI, kg/m^2	4.39 (2.55)	5.38 (2.75)	0.028*	135
SMM, kg	22.2 (6.04)	22.3 (6.66)	0.934	135
ASMM, kg	17.0 (4.27)	17.3 (4.44)	0.791	135
SMI, kg/m^2	8.07 (1.62)	8.05 (1.83)	0.805	135
MM, kg	22.2 (6.04)	22.3 (6.66)	0.934	135
ECW, kg	17.3 (3.42)	18.1 (3.43)	0.232	135
TBW, kg	34.4 (6.76)	34.7 (7.12)	0.875	135
ECW/TBW	0.53 (0.06)	0.51 (0.06)	0.062	135
Na/K	1.29 (0.28)	1.28 (0.24)	0.856	135

	Low adherence *N=58*	High adherence *N=77*	***P* value**	**N**
Hydragram®, %	73.8 (2.74)	73.9 (3.04)	0.537	135
Nutrigram®, mg/24h/m	684 (184)	660 (184)	0.369	135
**Muscle mass quality**				
RF-CSA, cm^2	3.29 (1.27)	3.09 (1.26)	0.252	134
RF-CIRC, cm	8.45 (1.20)	8.20 (1.51)	0.293	134
RF-X-axis, cm	3.65 (0.51)	3.52 (0.68)	0.225	135
RF-Y-axis, cm	1.05 (0.35)	0.99 (0.28)	0.307	135
L-SAT, cm	0.74 (0.49)	0.75 (0.43)	0.534	135
T-SAT, cm	1.24 (0.55)	1.38 (0.60)	0.247	105
S-SAT, cm	0.60 (0.31)	0.64 (0.32)	0.509	101
VAT, cm	0.38 (0.17)	0.37 (0.22)	0.546	63
**Functional parameters**				
Hand grip strength, kg	25.3 (10.9)	23.5 (8.73)	0.458	135
Up and Go, seg	7.01 (1.45)	7.94 (2.21)	0.007**	135

Data are expressed as mean ± standard deviations or percentage. Groups were divided according to the adherence to the nutritional supplement, being high adherent and low-adherent groups included in the study. A Shapiro-Wilks test was performed to decide between normal or non-normal. Asterisk indicates significant differences between groups according to the T-test or Mann Whitney test according to the normality of the variables and Chi squared test was used for variables expressed as percentage (***p<0.001, **p<0.01, *p<0.05). **Abbreviations**: BCM: Body cell mass; BCMI: BCM index; BMI: Body mass index; BIVA: Bioelectrical Impedance Vectorial Analysis; FM: Fat mass; FMI: FM index; FFMI: Fat-free mass index; PA: Phase angle; RF-CIR: circumference of quadriceps rectus femoris; RF-CSA: rectus femoris cross-sectional area; SAT: subcutaneous adipose fat of leg (L), superficial (S) and total (T) abdominal; SMI: Skeletal muscle index; SPA: Standardized phase angle.
