# Supplementary material for: Adherence to β-hydroxy-β-methylbutyrate-Enriched Oral Nutritional Supplements Enhances Survival and Nutritional Recovery in Malnourished Outpatients: Prognostic Insights
Source: Nutrients. 2025 May 7;17(9):1601. doi: 10.3390/nu17091601 (PMC12073151; doi:10.3390/nu17091601)
Supplement: Supplementary file 1 [file nutrients-17-01601-s001.zip › Supplementary Table S3.pdf]

Supplementary Table S2. Changes in Nutritional Status after Nutritional Intervention in the Low Adherence Group

**Variable**	**Baseline mean**	**SD**	**After mean**	**SD**	***p* value**	**Change (%)**	**Difference**
**Anthropometric variables**							
Weight, kg	58.241	13.160	56.828	13.295	0.002**	-2.435	-1.414
Body-mass index, kg/m²	21.383	4.042	20.857	4.094	0.002**	-2.409	-0.526
**BIVA variables**							
PA, º	5.140	1.069	4.624	1.027	0.000***	-9.480	-0.516
SPA	0.229	1.549	-0.493	1.653	0.000***		-0.722
BCM, kg	22.783	6.267	20.162	5.469	0.000***	-10.644	-2.621
BCMI, kg/m^2	8.293	1.855	7.403	1.725	0.000***	-10.113	-0.890
FFM, kg	46.374	8.907	44.598	8.539	0.000***	-3.654	-1.776
FFMI, kg/m^2	16.990	2.183	16.334	2.022	0.000***	-3.636	-0.655
FM, kg	11.867	6.759	12.229	7.696	0.386	4.984	0.362
FMI, kg/m^2	4.391	2.553	4.514	2.871	0.419	4.815	0.122
SMM, kg	22.197	6.042	21.424	6.627	0.041*	-3.624	-0.772
ASMM, kg	17.033	4.270	16.314	4.405	0.001*	-4.211	-0.719
SMI, kg/m^2	8.071	1.622	7.764	1.731	0.015*	-3.621	-0.307
MM, kg	22.197	6.042	21.424	6.627	0.041*	-3.624	-0.772
ECW, kg	17.338	3.415	18.088	5.201	0.075	3.695	0.750
TBW, kg	34.428	6.757	33.479	7.085	0.010*	-2.720	-0.948
Na/K	1.291	0.282	1.431	0.541	0.027*	11.304	0.140
Hydragram®, %	73.829	2.745	74.803	4.183	0.028*	1.328	0.974
Nutrigram®, mg/24h/m	684.350	184.426	609.238	163.230	0.000***	-10.132	-75.112
**Nutritional ultrasound**							
RF-CSA, cm^2	3.288	1.268	2.848	1.132	0.000***	-12.517	-0.439
RF-CIRC, cm	8.446	1.201	8.208	1.287	0.008**	-2.714	-0.239
RF-X-axis, cm	3.649	0.508	3.476	0.538	0.001**	-4.506	-0.174

Variable	Baseline mean	SD	After mean	SD	*p* value	Change (%)	Difference
RF-Y-axis, cm	1.052	0.351	0.886	0.277	0.000***	-13.678	-0.166
L-SAT, cm	0.745	0.495	0.719	0.514	0.437	-0.607	-0.026
T-SAT, cm	1.236	0.550	1.159	0.706	0.306	-6.213	-0.077
S-SAT, cm	0.601	0.307	0.547	0.406	0.720	0.438	-0.055
VAT, cm	0.375	0.174	0.421	0.356	0.011*	-17.898	0.046
**Functional parameters**							
Hand grip strength, kg	25.310	10.908	22.845	9.769	0.000***	-9.025	-2.466
Up and Go, s	7.012	1.451	7.852	1.740	0.000***	12.454	0.840

Data are expressed as mean ± standard deviations. A Shapiro-Wilks test was performed to decide between normal or non-normal. Asterisk indicates significant differences between groups according to the paired t-test or Wilcoxon test according to the normality of the variables (***p<0.001, **p<0.01, *p<0.05). **Abbreviations**: BCM: Body cell mass; BCMI: BCM index; BMI: Body mass index; BIVA: Bioelectrical Impedance Vectorial Analysis; FM: Fat mass; FMI: FM index; FFMI: Fat-free mass index; PA: Phase angle; RF-CIR: circumference of quadriceps rectus femoris; RF-CSA: rectus femoris cross-sectional area; SAT: subcutaneous adipose fat of leg (L), superficial (S) and total (T) abdominal; SMI: Skeletal muscle index; SPA: Standardized phase angle.
