# Supplementary figures and images for: Adherence to β-hydroxy-β-methylbutyrate-Enriched Oral Nutritional Supplements Enhances Survival and Nutritional Recovery in Malnourished Outpatients: Prognostic Insights
Source: Nutrients. 2025 May 7;17(9):1601. doi: 10.3390/nu17091601 (PMC12073151; doi:10.3390/nu17091601)

## Slide 1
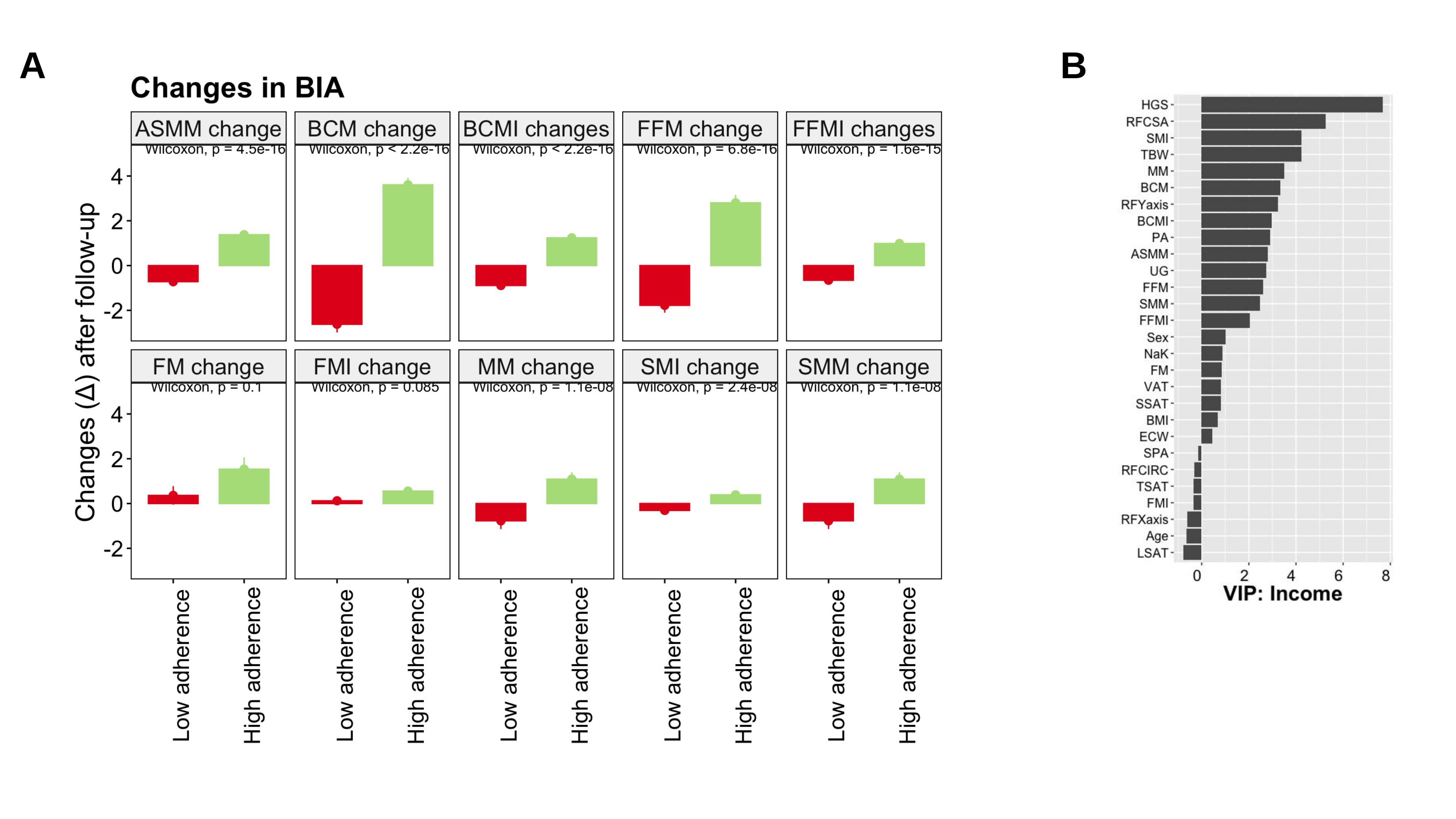

A
B

Supplement: Supplementary file 1 [file nutrients-17-01601-s001.zip › Supplementary Figure S2.pptx]

## Slide 1
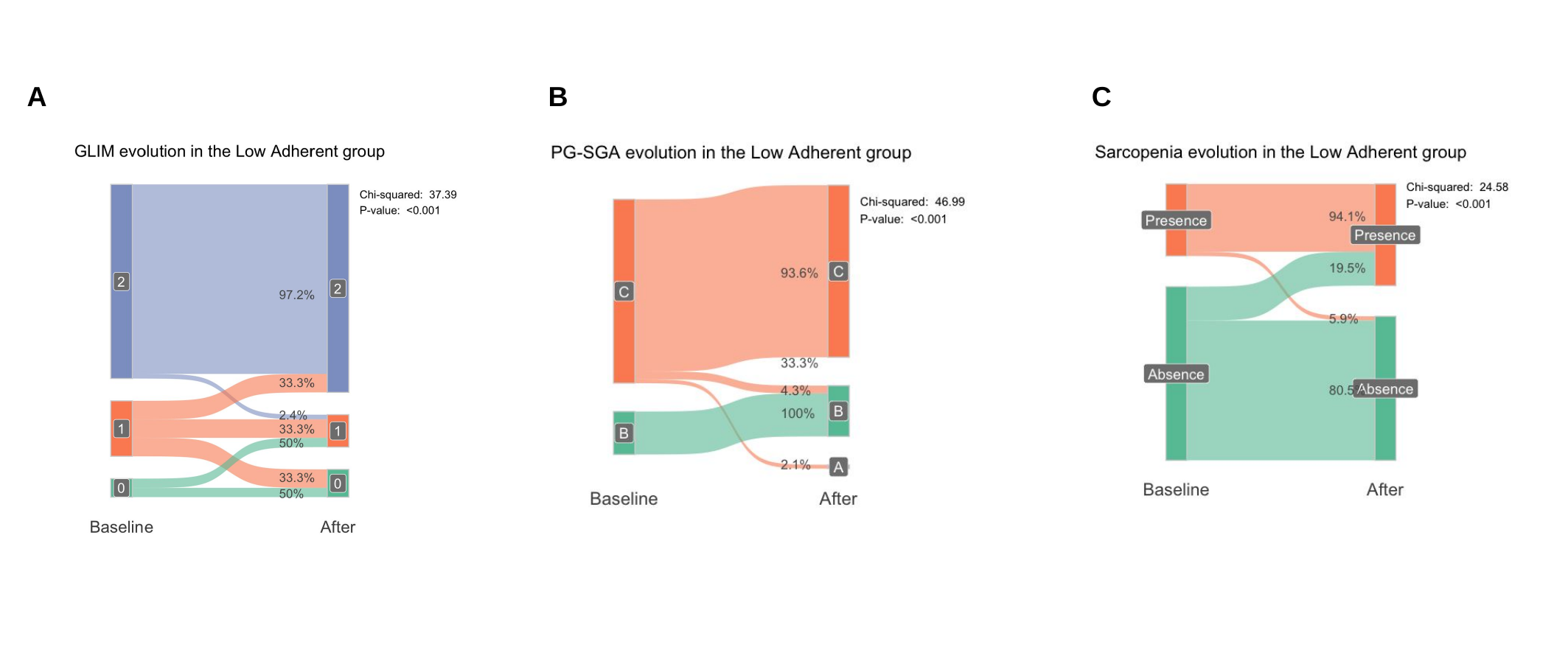

A
B
C

Supplement: Supplementary file 1 [file nutrients-17-01601-s001.zip › Supplementary Figure S3.pptx]

## Slide 1
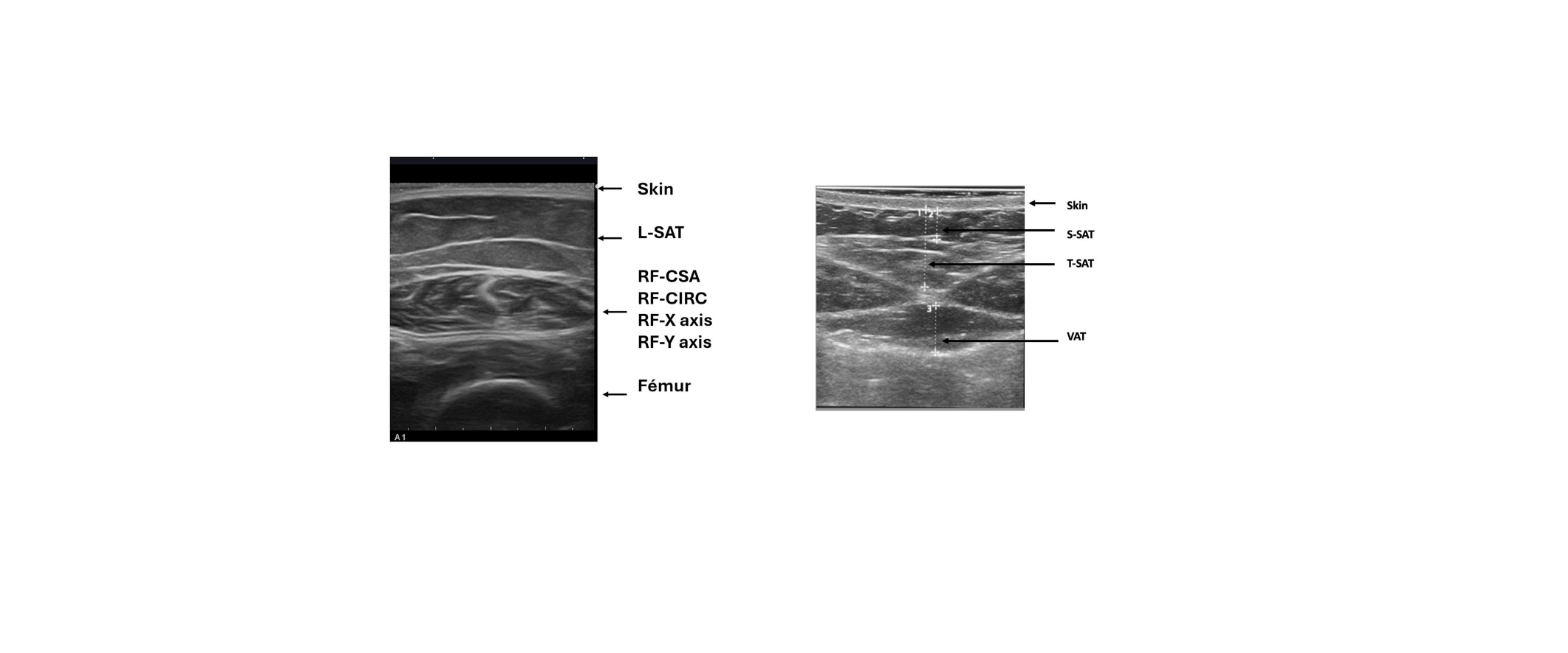

Supplement: Supplementary file 1 [file nutrients-17-01601-s001.zip › Supplementary Figure S4.pptx]
